# Correlation of SARS-CoV-2 in Wastewater and Individual Testing Results in a Jail, Atlanta, Georgia, USA

**DOI:** 10.3201/eid3013.230775

**Published:** 2024-04

**Authors:** Lindsay B. Saber, Shanika S. Kennedy, Yixin Yang, Kyler N. Moore, Yuke Wang, Stephen P. Hilton, Tylis Y. Chang, Pengbo Liu, Victoria L. Phillips, Matthew J. Akiyama, Christine L. Moe, Anne C. Spaulding

**Affiliations:** Emory University, Atlanta, Georgia, USA (L.B. Saber, S.S. Kennedy, Y. Yang, K.N. Moore, Y. Wang, S.P. Hilton, P. Liu, V.L. Phillips, C.L. Moe, A.C. Spaulding);; Zucker School of Medicine at Hofstra/Northwell, Hempstead, New York, USA (T.Y. Chang);; Albert Einstein College of Medicine, Bronx, New York, USA (M.J. Akiyama)

**Keywords:** COVID-19, wastewater-based surveillance, epidemiology, virus detection method, jail, correctional, SARS-CoV-2, viruses, coronaviruses, respiratory infections, zoonoses, Atlanta, Georgia, United States

## Abstract

Institution-level wastewater-based surveillance was implemented during the COVID-19 pandemic, including in carceral facilities. We examined the relationship between COVID-19 diagnostic test results of residents in a jail in Atlanta, Georgia, USA (average population ≈2,700), and quantitative reverse transcription PCR signal for SARS-CoV-2 in weekly wastewater samples collected during October 2021‒May 2022. The jail offered residents rapid antigen testing at entry and periodic mass screenings by reverse transcription PCR of self-collected nasal swab specimens. We aggregated individual test data, calculated the Spearman correlation coefficient, and performed logistic regression to examine the relationship between strength of SARS-CoV-2 PCR signal (cycle threshold value) in wastewater and percentage of jail population that tested positive for COVID-19. Of 13,745 nasal specimens collected, 3.9% were COVID-positive (range 0%–29.5% per week). We observed a strong inverse correlation between diagnostic test positivity and cycle threshold value (r = −0.67; p<0.01). Wastewater-based surveillance represents an effective strategy for jailwide surveillance of COVID-19.

Jails, which are short-term carceral institutions, experienced numerous factors during the COVID-19 pandemic that can lead to SARS-CoV-2 transmission, including crowding, mask shortages, and difficulty implementing sufficient quarantine and isolation practices (*1**,**2*). In 2020, 7% of US jails were operating over capacity, despite total admissions decreasing from 10.3 million in 2019 to 8.7 million in 2020 (16%) (*3*). Although the Centers for Disease Control and Prevention published guidelines for COVID-19 management in carceral settings (*4*), COVID-19 incidence exceeded that of surrounding communities up to 5-fold (*5*–*7*). In addition, there are logistical challenges to regularly screening jail residents for asymptomatic disease, especially in large jails that house thousands of persons (*5*,*8*–*12*).

Wastewater-based surveillance (WBS) might detect SARS-CoV-2 before onset of clinical symptoms and could serve as a sensitive, noninvasive early warning tool both regionally and at an institutional level (*13*–*19*). WBS might also limit biases that arise from residents avoiding testing or medical care. If implemented in jails, WBS could potentially save time, resources, and lives. This study examined WBS for monitoring SARS-CoV-2 infection in a large jail in Atlanta, Georgia, USA.

## Methods

### Setting and Population

The Emory University Institutional Review Board determined that this study constituted non–human subject research. The study was set in the Fulton County (Georgia) Jail, which has a 2,600-person capacity (*20*). The mean ± SD population during our study period, October 20, 2021–May 4, 2022, was 2,700 ± 133 persons. The main complex has north and south towers, each with 7 floors, and 6 housing units per floor. People entering move into housing units within 24 hours, predominantly to 1 designated floor of the south tower. Housing units typically hold 40 persons maximum, in 20 two-person cells. When volume exceeds capacity, residents sleep on mattresses on the floor. The population of this study was jail residents, who on average outnumber correctional officers 15-fold.

### Wastewater Monitoring

**Figure 1:**
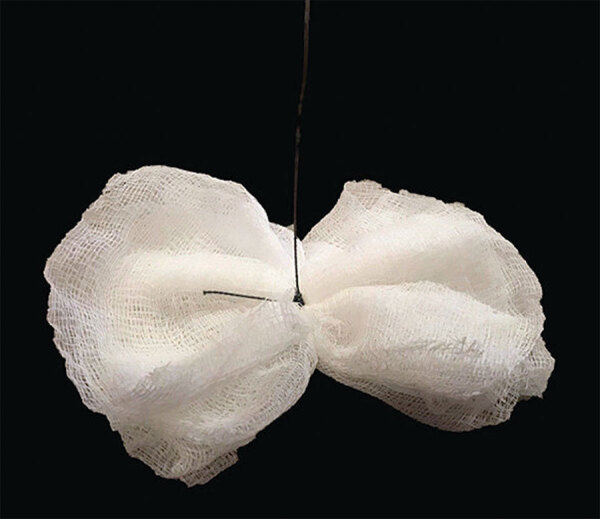
Moore swab: 4-in by 4-in cotton gauze squares tied together with nylon fishing line (*21*).

**Figure 2:**
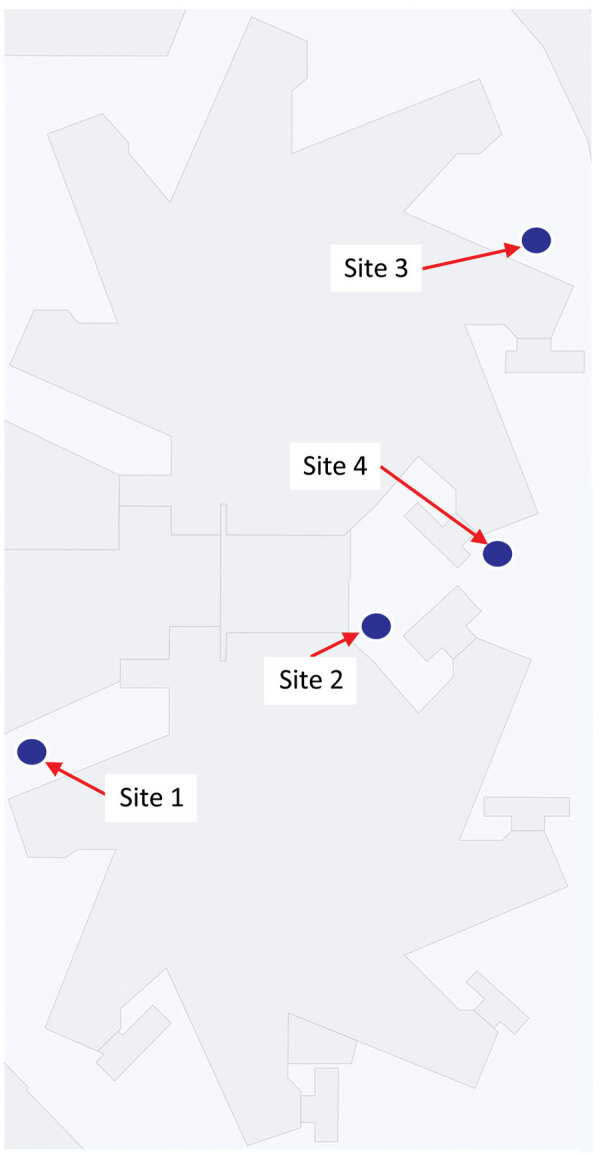
Outline of the Fulton County Jail, Atlanta, Georgia, USA, showing wastewater-based surveillance collection sites. Site 3 was used for final analysis as a proxy for wastewater-based surveillance results of the entire jail.

A sampling team from Emory University in Atlanta collected weekly wastewater samples from the jail throughout the project period. Moore swabs (Figure 1) were suspended overnight in manhole sites around the jail property (Figure 2) (*21*,*22*). Eluted wastewater from the swabs was tested by using quantitative real-time reverse transcription PCR (qRT-PCR) at the Center for Global Safe Water, Sanitation, and Hygiene Laboratory at Emory University, as described (*21*,*23*). The amount of SARS-CoV-2 viral RNA present in a sample was measured by qRT-PCR cycle threshold (Ct) value, which is inversely related to the concentration of SARS-CoV-2 in the Moore swab eluate. Positive samples were defined as those with qRT-PCR results in both duplicate wells <40 Ct and within 2 Ct of each other. For this analysis, wastewater data originated from a single, downstream collection point, site 3 (Figure 2), which contained a mixture of wastewater from the south and north towers (Appendix). We used those data as proxy for wastewater concentration of SARS-CoV-2 for the entire jail. Samples were not collected during 3 holiday weeks in November‒December 2021.

### COVID-19 Individual Diagnostic Testing

Healthcare staff routinely offered residents opt-out, rapid antigen testing at intake, as part of the jail’s entry protocol (BinaxNOW, Abbott Laboratories, https://www.abbott.com, through January 31, 2022; QuickVue, Quidel Corporation, https://www.quidel.com, starting February 1, 2022). After intake, antigen testing was available if residents exhibited COVID-19 symptoms, or upon request.

An Emory University team offered opt-out mass screening to a subset of jail residents on a weekly basis. Residents opting in provided self-collected nasal specimens, which were tested by RT-PCR. Insufficient staffing precluded offering mass screening to the entire resident population at any single timepoint. Each week, areas of the jail screened by RT-PCR were either randomly selected or targeted on the basis of known ongoing outbreaks. 

### Data Analysis

We stored, managed, and analyzed all data in Excel software (Microsoft, https://www.microsoft.com) and R software (The R Foundation for Statistical Computing, https://www.r-project.org). We aggregated the PCR data with results of intake antigen testing to calculate the percentage of diagnostic tests with positive results at each timepoint. First, we analyzed diagnostic test results and wastewater RT-PCR results separately to examine temporal trends. We then compared those trends through time-matched results from the COVID-19 diagnostic tests and WBS. We calculated the Spearman correlation coefficient (r) for the relationship between Ct values of wastewater samples and percentage of the COVID-19 diagnostic tests that had positive results. Last, we performed a logistic regression analysis of the presence or absence of SARS-CoV-2 in wastewater samples and the percentage of positive COVID-19 diagnostic tests matched by week. We assigned a Ct value of 40 when the RT-PCR result for a wastewater sample was negative.

## Results

The jail population during the study period ranged from 2,497 to 2,904 residents. Most (98.4%) persons in the jail during this period were male; 88.8% were Black. 

### Wastewater Monitoring

A total of 79 wastewater samples were collected from 4 manhole sites (Figure 2). Spearman correlation coefficients showed strong correlations between Ct values of wastewater samples collected from different sites on the same day (Appendix Table 2, Figure 2), confirming that results from 1 site (site 3) sufficed as a jailwide proxy.

SARS-CoV-2 was detected in 20 (80%) of 25 Moore swab samples of wastewater from site 3 during the study period. Of the 20 positive samples in the study period, the mean Ct value was 33.94 (SD 3.74). 

**Figure 3:**
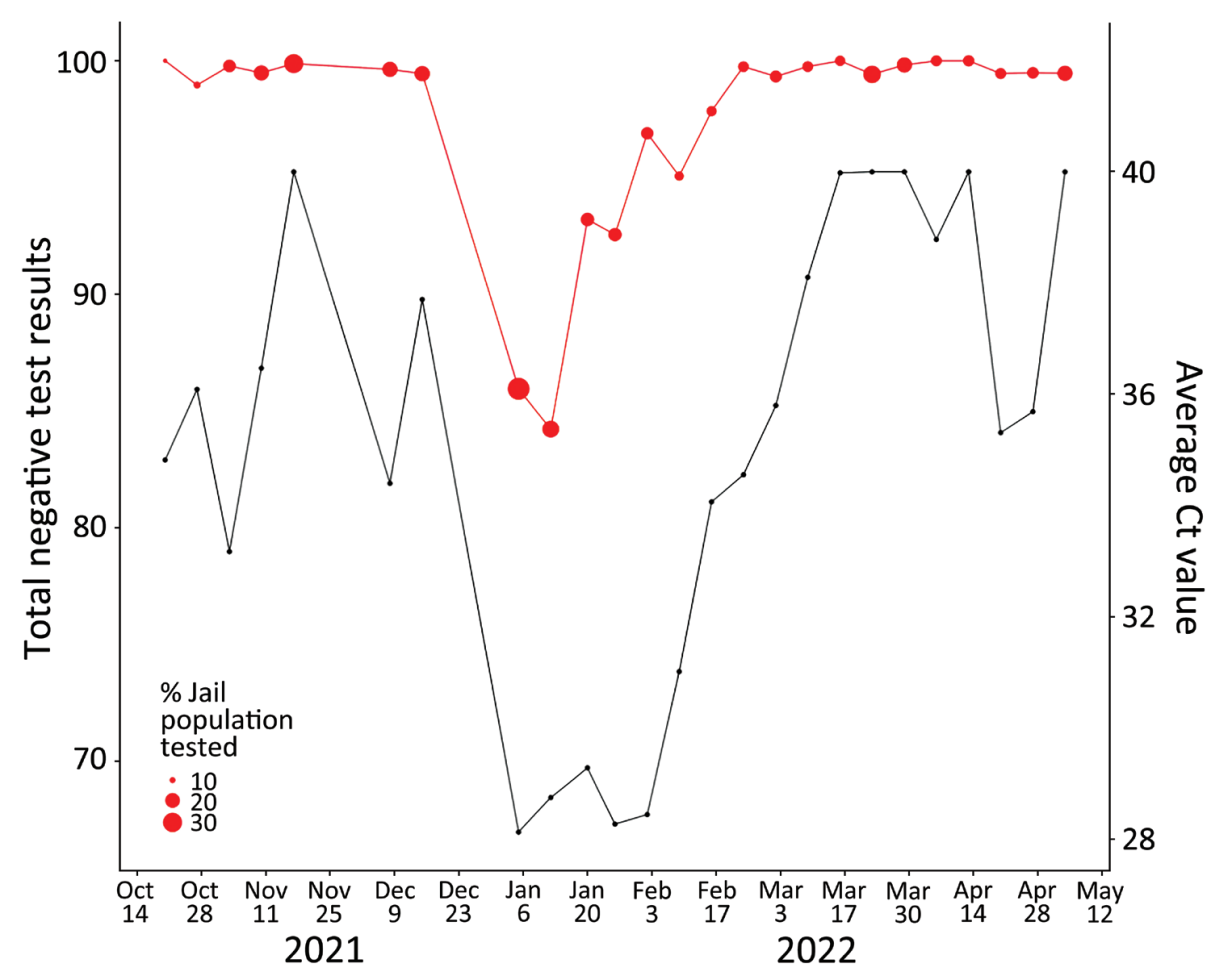
Average Ct values of wastewater samples (black lines) versus total percentage of negative COVID-19 diagnostic test results (red lines), Fulton County Jail, Atlanta, Georgia, USA, October 2021‒May 2022. Dot sizes are proportional to the percentage of the jail population undergoing a COVID-19 diagnostic test for the corresponding week. Ct, cycle threshold.

There was considerable temporal variability in the wastewater Ct values during the study period (Figure 3). The wastewater Ct value decreased sharply between the samples collections during the week of December 15, 2021, and during the week of January 5, 2022. This decrease was followed by the lowest Ct value during the study period (28.1 on January 5, 2022, which was during the Omicron virus surge in Atlanta). The wastewater Ct values were in that range for 5 consecutive weeks of the surge (Figure 3). No SARS-CoV-2 RNA was detected in wastewater samples from 1 sampling date in November 2021 or from 4 sampling dates in March‒April 2022.

### COVID-19 Diagnostic Testing

A total of 17 mass diagnostic PCR testing events resulted in 3,770 total self-collected swab specimens tested by RT-PCR. A total of 9,975 rapid COVID-19 diagnostic tests were conducted at intake over 28 weeks (Table 1).

**Table 1:** Demographic characteristics of residents in the Fulton County Jail Main Complex, Atlanta, Georgia, USA, October 20, 2021‒May 4, 2022*

Characteristic	% Jail population
Reported sexual assignment	
M	98.4
F	1.6
Race/ethnicity	
Black, non-Hispanic	88.8
White, non-Hispanic	10.3
Hispanic	<1
Other	<1
Charges	
Misdemeanor only	6.8
Felony	93.2

*Source: Fulton County Jail.

**Figure 4:**
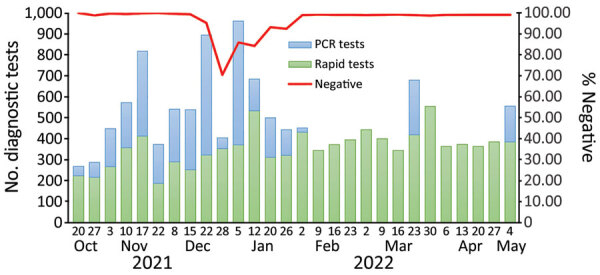
PCR and rapid COVID-19 diagnostic test results, Fulton County, Jail, Atlanta, Georgia, USA, October 2021‒May 2022. The percentage of the combined negative diagnostic results is overlaid, showing peak in positive results (i.e., nadir of negative results) in late December 2021.

The median number of diagnostic tests conducted each week was 443 (Table 2). Most were rapid antigen tests (median = 363) rather than PCR diagnostic tests (median = 186). The weekly percentage positivity for PCR tests and for rapid antigen tests were highly correlated (r = 0.91) (Table 3). We aggregated the PCR test and rapid antigen test results to calculate the weekly diagnostic test positivity rate during October 20, 2021–May 4, 2022. The combined test positivity averaged 3.9% (SD 6.6%) over the study period. We compiled the number of weekly COVID-19 diagnostic tests administered and the percent positivity over the study period (Figures 3, 4). The percentage positivity fluctuated but increased as the study progressed (Figure 3).

**Table 2:** Summary of COVID-19 diagnostic testing results at the Fulton County Jail, Atlanta, Georgia, USA, October 2021–May 2022*

COVID-19 diagnostics weekly	Mean (SD) per week	Median per week	Minimum–maximum per week	Totals over entire study
No. diagnostic tests	491 (176)	443	267–961	13,745
No. rapid tests	356 (84)	363	186–554	9,975
No. PCR tests	222 (167)	186	20–591	3,770
% Jail population tested†	18.3 (7.1)	16	9.7–38.2	NA
Overall percentage positivity‡	3.39 (6.56)	0.55	0–29.5	NA

*NA, not applicable. †Numerator is the number of positive test results in a given week, denominator is the jail population for the week. ‡Numerator is the number of positive test results in a given week, denominator is the total tests for the same week

**Table 3:** Spearman correlation coefficients (r) for percentage positivity of diagnostic tests and wastewater Ct values within and between variable groupings, Fulton County Jail, Atlanta, Georgia, USA, October 20, 2021‒May 4, 2022*

Diagnostic test	r (p value)		
	PCR	Rapid antigen	Total
PCR	Referent	0.91 (<0.01)	0.78 (<0.01)
Rapid antigen		Referent	0.97 (<0.01)
Total			Referent
Wastewater and diagnostic correlation¶			
Wastewater Ct values	−0.54 (0.048)	−0.64 (<0.01)	−0.67 (<0.01)

*Percentage positivity for each category was computed by dividing the number of positive test results in 1 week by the total number of tests administered for the same week. Each datapoint is correlated with all other datapoints; none are grouped based on date or other variables. Ct, cycle threshold. †Based on wastewater Ct values.

PCR tests consistently had a higher percent positivity than the routine rapid antigen tests. During the midwinter surge, there was a much higher proportion of positive PCR tests (e.g., week of December 28, 2021, 63.5%) compared with positive rapid antigen test results (24.4%). Nonetheless, the positivity rates for the PCR test and rapid antigen test were correlated during weeks when both tests were administered (r = 0.65; p = 0.004).

### Wastewater and Diagnostic Comparison

When the percent positivity for diagnostic tests was low for several weeks (e.g., March 9, 2022–April 13, 2022), the Ct values for the wastewater samples were high (38.1‒40) or negative, indicating little or no detection of SARS-CoV-2 RNA in the wastewater samples. Low Ct values were measured in the wastewater samples during the weeks when the COVID-19 diagnostic test percent positivity was high (e.g., early January 2022). Overall, the total COVID-19 diagnostic test percent positivity had a strong negative correlation with the wastewater Ct values combined over time (r = −0.67; p<0.01) (Table 3).

We used logistic regression to analyze the relationship between percent positivity in diagnostic testing and WBS results as a dichotomous outcome (presence/absence of SARS-CoV-2). Holding all other predictors constant, we found that the odds of a positive WBS reading increased by 4.773 (95% CI 3.701–5.845) for each percentage point increase in diagnostic test percent positivity (Appendix Figure 3).

## Discussion

Percent positivity of COVID-19 diagnostic testing among jail residents correlated with SARS-CoV-2 detection in the jail wastewater during the same time periods, which provides evidence that WBS can serve as an indicator of viral infection within the jail. The study team’s inability to gather self-collected specimens from all jail residents in a single week supports the need for an aggregate indicator of population infection. Overall, our data indicate that WBS was a sensitive signal for COVID-19 cases in the jail population and of surges in infection (*6*,*10*,*24*,*25*).

The experience in this jail indicates that WBS can detect the beginning of an outbreak before clinical signs appear. The spike in COVID-19 cases in the jail (January 5, 2022) occurred 8 days before a community surge in Fulton County and aligned with COVID-19 case surges in Atlanta and nationwide caused by the Omicron variant (*24*). Therefore, jails might serve as an early warning signal for community spikes for COVID-19 and other infectious diseases detectable in wastewater. This study also demonstrated the efficiency and feasibility of conducting WBS for SARS-CoV-2 on a regular basis in a jail setting. Although the median number of rapid (n = 363) and PCR (n = 186) tests differed during the study period, the strong correlation between the positivity rate of the 2 different tests (r = 0.65; p<0.01) suggests relatively accurate results from both forms of diagnostic tests. Over the fall of 2021, the portion of the jail population that participated in the mass testing events (Figure 3) trended upward because of efficiencies introduced (Appendix).

As previous WBS studies on university campuses have noted, collecting and processing a few Moore swab samples in this study was faster and much less expensive than individual diagnostic testing of all jail residents (*26*). Because of this finding, there are still several functioning WBS programs, with potential to expand to other infectious diseases. A report on costs of WBS in this study is pending. Future work will examine the use of WBS to detect other pathogens present in the jail population, and possibly sequencing COVID-19 strains that are detected in the wastewater to contribute to molecular surveillance.

Strengths of this study include sufficient numbers of diagnostic tests and WBS samples to enable weekly comparisons between the 2 testing methods, and close collaboration with jail officials that provided the opportunity to conduct the study over a full 6-month period that captured temporal trends, including the entirety of the Omicron variant peak. Over the fall of 2021, the portion of the jail population that participated in the mass testing events for this study (Figure 3) trended upward because of efficiencies introduced (Appendix).

The first limitation of this study is that jail size precluded diagnostic testing of the entire jail population in any single week; percentage positivity from the portion tested for COVID-19 was used as a proxy. In addition, individual PCR tests were run outside of mass testing events as needed for the purposes of the jail’s infection control program, not conducted simply for populationwide surveillance. Nonetheless, testing was never confined to jail areas known to have high or low COVID-19 prevalence. Second, the qRT-PCR results (Ct values) for the Moore swab samples are a semiquantitative indicator of SARS-CoV-2 concentration in wastewater because of the unknown volume of wastewater that passes through the swab (*16*). Third, a jail is not a closed system; many residents enter and leave daily. A resident who sheds fecal matter containing SARS-CoV-2 might leave the jail before the next round of individual COVID-19 screening and would therefore only be represented in the wastewater results. Fourth, only COVID-19 tests among residents were included in our analyses. However, because there are ≈15 times as many jail residents than staff, fecal material from staff probably had a negligible effect on the WBS results.

WBS was an efficient and accurate approach for tracking trends in SARS-CoV-2 infection in this jail population. Its most useful role might be as a sentinel surveillance tool when the signal switches from negative to positive, indicating a need for diagnostic testing in specific areas of the jail. Even under ideal circumstances with adequate resources, administering individual weekly COVID-19 diagnostic tests to the entire Fulton County Jail was not a feasible COVID-19 surveillance strategy. The WBS results aligned well with the percentage positivity of COVID-19 diagnostic tests among jail residents and could serve as a sensitive and economical surveillance tool for COVID-19 for this jail. In addition, because residents of the jail come from a wide geographic range in a large county, our results suggest that WBS at the jail could be useful for understanding COVID-19 trends in the jail itself to guide primary prevention and response to mitigate transmission and that jails could serve as a valuable sentinel site for monitoring trends in COVID-19 cases and genetic variants in the wider community.

Appendix Correlation of SARS-CoV-2 in wastewater and individual testing results in a jail, Atlanta, Georgia, USA.
