# Supplementary material for: Correlation of SARS-CoV-2 in Wastewater and Individual Testing Results in a Jail, Atlanta, Georgia, USA
Source: Emerg Infect Dis. 2024 Apr;30(Suppl 1):S21–7. doi: 10.3201/eid3013.230775 (PMC10986836; doi:10.3201/eid3013.230775)
Supplement: Appendix — Correlation of SARS-CoV-2 in wastewater and individual testing results in a jail, Atlanta, Georgia, USA. [file 23-0775-Techapp-s1.pdf]

Article DOI: https://doi.org/10.3201/eid2813.230775

EID cannot ensure accessibility for Supplemental Materials supplied by authors. Readers who have difficulty accessing supplementary content should contact the authors for assistance.

Correlation of SARS-CoV-2 in Wastewater and Individual Testing Results in a Jail, Atlanta, Georgia, USA

Appendix

Comparison of Wastewater-Based Surveillance in Jails versus University Dormitories

Wastewater-based surveillance (WBS) has been studied in non-carceral institutions, such as university residence halls, where the turnover rate of those who reside in the dorm is low, and students normally live an entire semester in the same room but can have frequent visitors (*1–6*). In contrast, the median length of stay for a Fulton County Jail resident has historically been 5 days, and the mean stay is 22 days (*7*). This contrast between residence times in university dormitories versus a jail motivates a study specifically for the jail setting. There may be different utility of WBS data in jails compared with other settings.

Dye Testing at Fulton County Jail to Trace Flow of Wastewater

For this study, we first assumed that the source of wastewater flowing under each manhole corresponded to only the zone of the building in closest proximity to the collection point. This assumption was tested by adding tracer dye (EcoClean Solutions, Copiague NY) into toilets in various housing blocks of the jail. When the dye from one housing block was detected in wastewater at multiple manholes, we concluded that the sewerage lines from multiple housing units were connected and that a wastewater sample from one manhole collection point could represent multiple housing blocks. Dye testing indicated that wastewater from both towers accumulated in Site 3 (See Figure 2, main manuscript), and the results from this site were used as a proxy for the wastewater concentration of SARS-CoV-2 for the entire jail.

COVID-19 Individual Diagnostic Testing: PCR Nasal Swab Collection

Concurrent with initiating WBS, a team from Emory University began a mass diagnostic screening program in October 2021. We piloted self-collection of nasal specimens for PCR

testing by the jail residents by using a SteriPack swab [SteriPack, Lakeland FL], following a previously reported qualitative study of the acceptability of this novel specimen collection strategy (*8*). On dates when mass collection of nasal swabs was performed, we hired off-duty correctional officers to escort the Emory specimen collection teams to areas of the jail to distribute and retrieve the nasal swabs. Bar codes, pre-printed on the tubes, were scanned into a cloud-based registration portal system for the diagnostic testing laboratory (Northwell Health Laboratory, Lake Success, NY) and minimized the time from specimen collection to registration to less than a minute per swab. The direct scanning of barcodes into the portal also minimized human error by eliminating typing specimen codes into the portal. We shipped swabs overnight to the laboratory for RT-PCR analysis by an LGC, Biosearch Technologies SARS-CoV-2 ultra-high-throughput End-Point RT-PCR Test (BT-SCV2-UHTP-EP) to detect positive nasal swabs (Biosearch Technologies, Hoddesdon, UK).

During the fall of 2021, there was an upward trend in the portion of the jail population that participated in the mass testing events (Figure 3). This occurred by establishing an efficient screening method and improving our collection routine over time so that each swab could be gathered and registered in under a minute. The jail's housing configuration (cells rather than open dormitories) slowed the collection process. Nonetheless, we demonstrated that our process could achieve specimen collection from multiple housing units per hour.

References

1. Wang Y, Liu P, Zhang H, Ibaraki M, VanTassell J, Geith K, et al. Early warning of a COVID-19 surge on a university campus based on wastewater surveillance for SARS-CoV-2 at residence halls. Sci Total Environ. 2022;821:153291. PubMed https://doi.org/10.1016/j.scitotenv.2022.153291

2. Karthikeyan S, Nguyen A, McDonald D, Zong Y, Ronquillo N, Ren J, et al. Rapid, large-scale wastewater surveillance and automated reporting system enable early detection of nearly 85% of COVID-19 cases on a university campus. mSystems. 2021;6:e0079321. PubMed https://doi.org/10.1128/mSystems.00793-21

3. Betancourt WQ, Schmitz BW, Innes GK, Prasek SM, Pogreba Brown KM, Stark ER, et al. COVID-19 containment on a college campus via wastewater-based epidemiology, targeted clinical testing and an intervention. Sci Total Environ. 2021;779:146408. PubMed https://doi.org/10.1016/j.scitotenv.2021.146408

4. Gibas C, Lambirth K, Mittal N, Juel MAI, Barua VB, Roppolo Brazell L, et al. Implementing building-level SARS-CoV-2 wastewater surveillance on a university campus. Sci Total Environ. 2021;782:146749. PubMed https://doi.org/10.1016/j.scitotenv.2021.146749

5. Liu P, Ibaraki M, VanTassell J, Geith K, Cavallo M, Kann R, et al. A sensitive, simple, and low-cost method for COVID-19 wastewater surveillance at an institutional level. Sci Total Environ. 2022;807:151047. PubMed https://doi.org/10.1016/j.scitotenv.2021.151047

6. Moody J. How Long Is a College Semester [cited 2023 Aug 23]. https://www.usnews.com/education/best-colleges/articles/how-long-is-a-college-semester

7. Spaulding AC, Kim MJ, Corpening KT, Carpenter T, Watlington P, Bowden CJ. Establishing an HIV screening program led by staff nurses in a county jail. J Public Health Manag Pract. 2015;21:538–45. PubMed https://doi.org/10.1097/PHH.0000000000000183

8. González-Montalvo MD, Dickson PF, Saber LB, Boehm RA, Phillips VL, Akiyama MJ, et al. Opinions of former jail residents about self-collection of SARS-CoV-2 specimens, paired with wastewater surveillance: a qualitative study rapidly examining acceptability of COVID-19 mitigation measures. PLoS One. 2023;18:e0285364. PubMed https://doi.org/10.1371/journal.pone.0285364

Appendix Table 1. Descriptions of variables used for analysis of test results, Fulton County Jail, October 5, 2021 – May 4, 2022.

Variable	Description
PCR tests	Those tests in which SARS-CoV-2 virus was detected.
Jail population	The total resident population of the Fulton County Jail determined through the resident count on weeks that the study team received rosters from jail personnel (point count of the jail). On the weeks that no rosters were received, public records from the Georgia Department of Community Affairs (DCA) were used to estimate the jail population (Georgia Department of Community Affairs, 2022).
Positivity rate	The total number positive diagnostic tests (PCR plus rapid) in a week divided by the total number of tests administered in the same week.
Percent of jail tested	The total number of diagnostic tests (PCR plus rapid) administered in a week divided by the jail population in the same week.
Ct Value	RT-qPCR cycle threshold value measured in wastewater samples at the Emory laboratory by using primers and probes for the N1 gene of SARS-CoV-2

Appendix Table 2. Spearman correlation coefficients of all Ct value wastewater results between 4 manhole sites. All values were statistically significant (α <0.05), October 2021–May 2022.

Site	Manhole 1	Manhole 2	Manhole 3	Manhole 5
Manhole 1	1			
Manhole 2	0.7437932	1		
Manhole 3	0.8324621	0.8945793	1	
Manhole 5	0.7075317	0.6935212	0.8041958	1

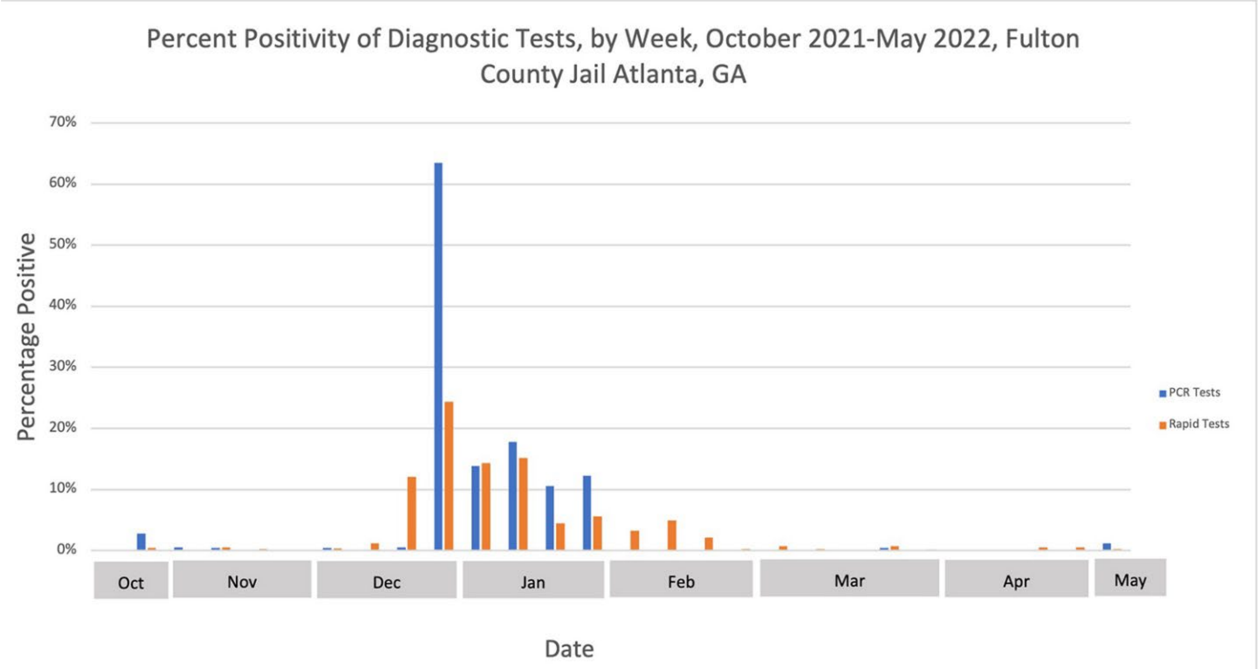

Appendix Figure 1. Percent positivity of both PCR and rapid diagnostic tests between October 2021–May 2022 at the Fulton County (Georgia) Jail.

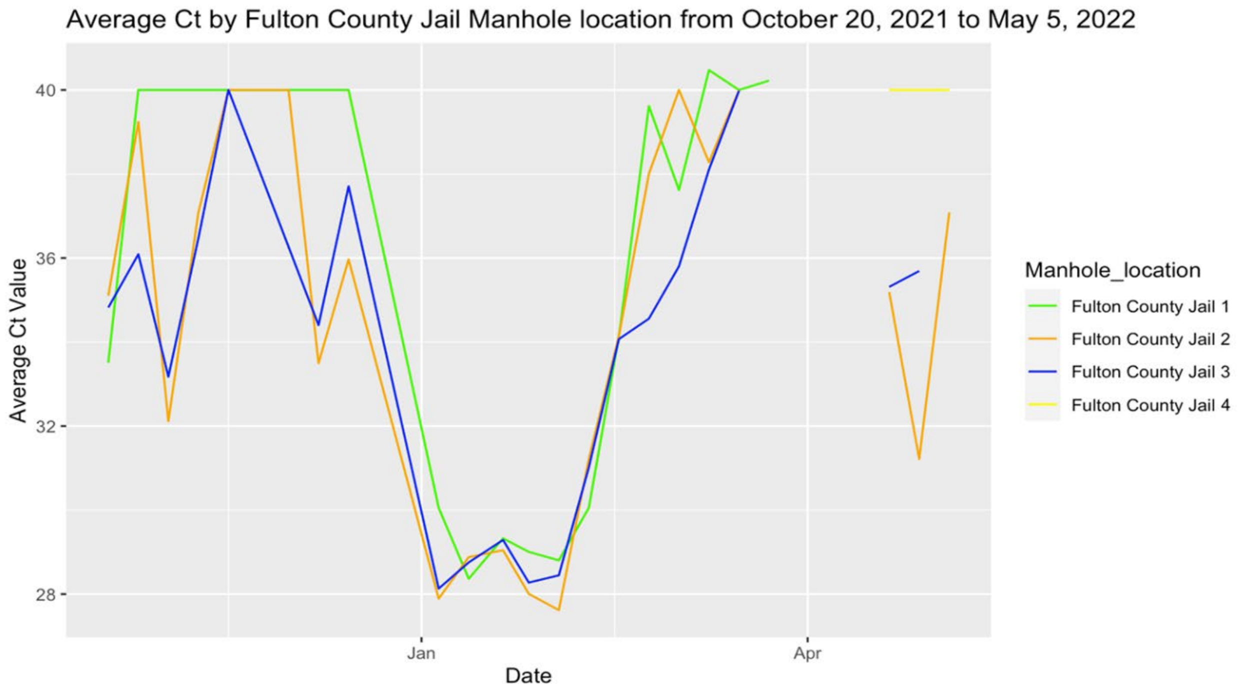

Appendix Figure 2. Average Ct value of the four manholes for wastewater collection between October 2021–May 2022 at the Fulton County (Georgia) Jail. Final analysis used only site 3, due to high correlation between results from site 1, 2, and 4 with site 3.

$$log\left(\frac{p}{1-p}\right) = \beta_0 + \beta_1 X,$$

Appendix Figure 3. Logistic regression of diagnostic test percent positivity and wastewater as a dichotomous outcome (positive or negative), Fulton County (Georgia) Jail, October 2021–May 2022. Where p is the probability of detecting SARS-CoV-2 in the wastewater sample from site 3 and X is the percent of positive COVID-19 diagnostic tests, the estimates of β0 and β1 were 0.484 and 4.773, respectively.

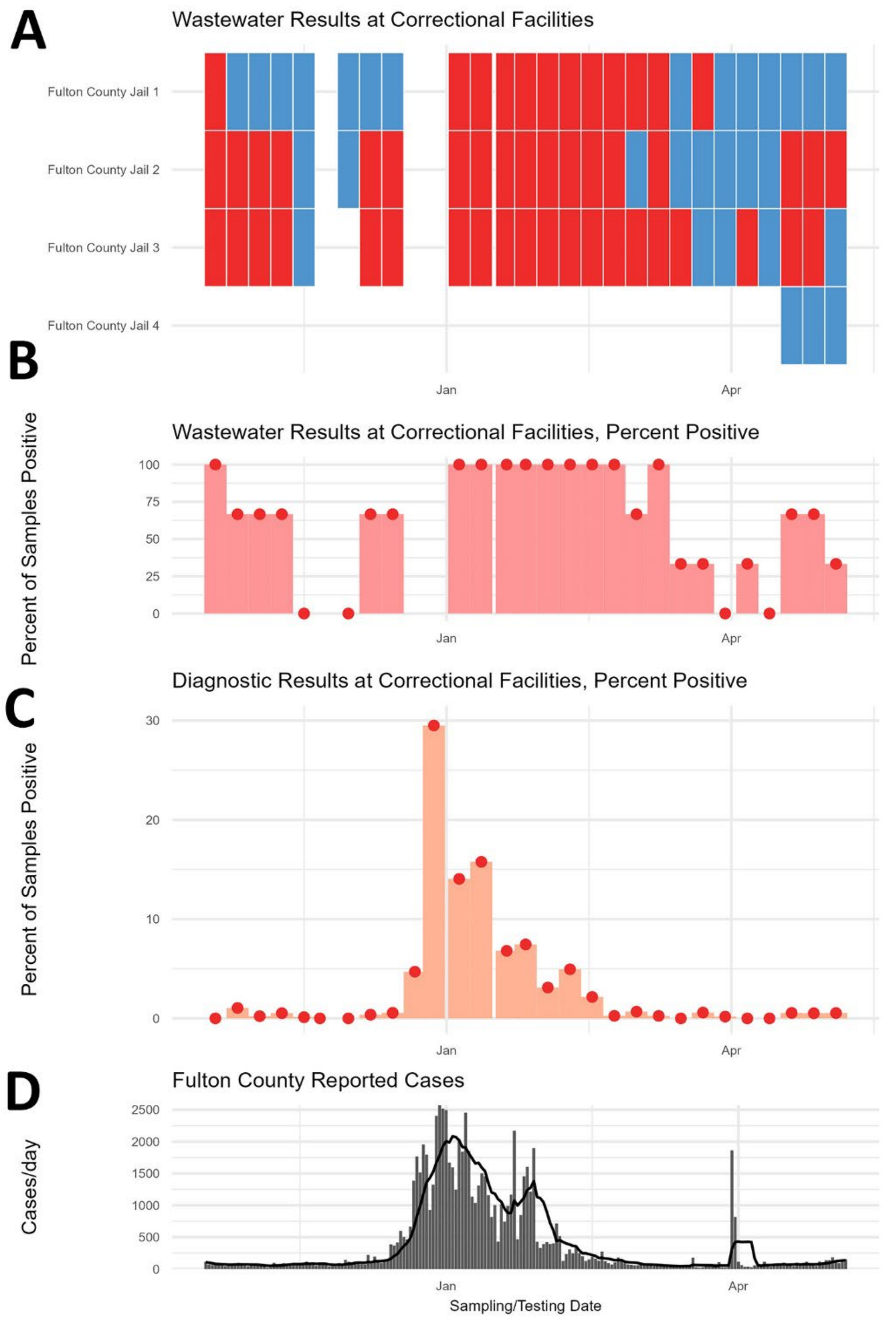

Appendix Figure 4. SARS-CoV-2 detected in wastewater at the Fulton County (Georgia) Jail, in diagnostic tests at the correctional facility, and in Fulton County from October 2021–May 2022. A) Dichotomous outcome of wastewater monitoring from four selected sampling point in the correctional facility, with blue tiles indicating a negative sample, red tiles indicating a positive sample, and any text representing average Rt-PCR Ct values. B) Percentage of positive wastewater samples from all sampling points in the correctional facility. Areas with a dot indicate that samples were collected that week. C) COVID-19 diagnostic test positivity rates. Areas with a dot indicate that samples were collected that week. D) Total reported cases in Fulton County, Georgia, with the line representing the 7-day average.
